# Evaluation of Effectiveness of a Novel Multicomponent Mycotoxins Detoxification Agent in the Presence of AFB1 and T-2 Toxin on Broiler Chicks

**DOI:** 10.3390/microorganisms11030574

**Published:** 2023-02-24

**Authors:** Darko Stefanović, Darko Marinković, Saša Trailović, Marko Vasiljević, Hunor Farkaš, Jog Raj, Nataša Tolimir, Stamen Radulović, Vladimir Nešić, Jelena Nedeljković Trailović, Branko Petrujkić

**Affiliations:** 1 Faculty of Veterinary Medicine, University of Belgrade, Bulevar Oslobodjenja 18, 11000 Belgrade, Serbia; 2 Patent Co., DOO, Vlade Ćetkovića 1A, 24211 Mišićevo, Serbia; 3 Institute for Science Application in Agriculture, 11000 Belgrade, Serbia

**Keywords:** AFB1, T-2 toxin, broiler chicks, health status, detoxification agent

## Abstract

This experimental study was conducted to determine the ability of a novel mycotoxins detoxification agent (MR) at a concentration of 0.2% to reduce the toxicity of aflatoxin B1 (AFB1) or T-2 toxin, alone or in combination, and to examine its effect on performance, pathohistological changes (PH) and the residue of these toxins in the tissues of broiler chicks. A total of 96 broiler chicks were divided into eight equal groups: group C, which served as control (without any additives); group MR, which received the novel detoxification agent (supplemented with 0.2%); group E-I (0.1 mg AFB1/kg of diet); group E-II (0.1 mg AFB1/kg of diet + MR 0.2%); group E-III (0.5 mg T-2 toxin/kg of diet); group E-IV (0.5 mg T-2 toxin/kg of diet + 0.2% MR); group E-V (combination of 0.1 mg AFB1/kg, 0.5 mg T-2 toxin/kg of diet); and group E-VI (combination of 0.1 mg AFB1/kg, 0.5 mg T-2 toxin + 0.2% MR). Results indicate that feeds containing AFB1 and T-2 toxin, alone or in combination, adversely affected the health and performance of poultry. However, the addition of MR to diets containing AFB1 and T-2 toxin singly and in combination exerted a positive effect on body weight, feed intake, weight gain, feed efficiency and microscopic lesions in visceral organs. Residual concentration of AFB1 in liver samples was significantly (*p* < 0.05) decreased when chicks were fed diets supplemented with 0.2% of MR.

## 1. Introduction

Mycotoxins are toxic secondary metabolites produced by some species of filamentous fungi, mainly those belonging to genera *Aspergillus*, *Alternaria*, *Fusarium*, *Claviceps* and *Penicillium* [1]. These fungi infect a variety of crops at the field level (preharvest) and/or during storage under favorable environmental conditions (postharvest), thereby causing severe reduction in yield and damage to the quality of the crop [2]. Nevertheless, many of these fungi produce several mycotoxins simultaneously, and some mycotoxins are produced by more than one fungal species [3]. Thus, it is not surprising that cereal grains and other crop species represent blends of different mycotoxigenic fungi species, which can grow on food and feeds simultaneously and are capable of producing more than one mycotoxin [4]. Such a contamination of feed/commodity by ‘mixture of mycotoxins’, usually at low concentrations, will result in increased exposure for humans and animals [5,6,7]. Consequently, health effects and combined toxic effects due to additive, synergistic or even antagonistic effects of mycotoxins can occur at different levels of exposure [8].

The contamination of feed and food materials with mycotoxins is a worldwide problem, but it is more predominant in regions affected by climatic conditions that favor the growth of fungi and production of mycotoxins [9]. As reported in several national, regional and worldwide mycotoxin surveys, approximately 90% of feed samples were contaminated with more than 10 mycotoxins, and about 75% of the samples contained at least 20 mycotoxins [8]. Although 400 potentially toxic secondary metabolites are known, only about 50 of them have been studied in detail due to their known toxicity and role played in food safety. The most important agro-economic and public health classes of mycotoxins, in terms of their prevalence and negative effects on human and animal health and performance, are (AFB1), ochratoxin A (OTA), zearalenone (ZEA), trichothecenes (TCT), fumonisins (FUMs), patulin (PT), alternaria toxins and ergot toxins [10]. However, the regulations worldwide do not take into account the combined effects of co-occurring mycotoxins [11].

Nowadays, particularly in southern Europe, AFB1 contamination has become increasingly significant as a consequence of rising average temperatures due to climate change, and it is expected to increase further. AFB1 are difuranocoumarin derivatives produced by a polyketide pathway by many strains of *Aspergillus flavus* and *Aspergillus parasiticus*, as well as the rare *Aspergillus nomius*, which contaminate agricultural commodities [12]. AFB1 is identified as the most potent naturally occurring carcinogen in this group, which can cause serious health issues, such as growth retardation, genotoxic, carcinogenic and teratogenic effects, with the liver being the primarily affected organ for both humans and animals [13]. IARC (2012) [14] classify AFB1, B2, G1, G2 and M1 as Group 1 carcinogens, emphasizing their explicit carcinogenicity to humans and animals. The non-observed adverse effect level is not applied for genotoxic carcinogens; therefore, no threshold is assigned to AFB1. Several studies have reported the negative effects of AFB1 on birds including a lower feed consumption rate, reduced weight gain due to a poor feed conversion rate, low egg production, and impaired resistance to infective diseases due to pathological damage to the immune-related organs, leading to increased mortality [15,16].

Other mycotoxins of great importance to livestock are TCT. The TCT mycotoxins comprise a vast group of more than 100 fungal metabolites with the same basic structure, affecting several major cereal crops including oats, barley, maize and wheat.

Poultry is quite sensitive to T-2/HT-2 toxins [17]. It is well known that DON and T-2 toxin are potent DNA protein synthesis inhibitors, and this can even lead to the disruption of DNA and RNA synthesis. A TCT-contaminated diet has been found to alter intestinal morphology, resulting in villi atrophy and reduced villi height [18]. In addition, studies have shown that TCT have a negative effect on the viability of intestinal cells and induce inflammation and oxidative stress in intestinal epithelial cells, thus accelerating cell apoptosis [19,20]. Furthermore, T-2 and its derivate HT-2 cause digestive disorders, oral lesions, immunologic effects and hematological disorder [21]. Ultimately, these changes in intestinal epithelial cells lead to an increase in intestinal permeability and reduced nutrition absorption, which result in poor performance in growth and expose animals to infectious diseases. Taken together, it is clear that mycotoxin contamination of poultry feed is an important animal health issue, as well as being responsible for significant economic loss for poultry producers [18,22,23].

Poultry feed is a mixture of various ingredients, which are susceptible to pre- and postharvest fungal infection, and this may lead to contamination with multiple mycotoxins. Therefore, the number of research studies analyzing multiple mycotoxins has increased [24,25]. To minimize the impact of mycotoxins on animal health and to reduce economic losses, different approaches including chemical, physical and biological methods have been adopted in the decontamination of poultry feeds [26]. Microbial degradation of mycotoxins is considered an effective means of mycotoxin remediation [27,28]. In addition, various strains of *Bacillus* sp. are utilized by the feed industry due to their positive probiotic effects in the gastrointestinal tract [28]. The efficacy of several adsorbents such as modified clinoptilolite in poultry feed decontamination have been extensively studied, but most of the studies are focused on only one or two mycotoxins such as aflatoxin and/or ochratoxin [29,30] or T-2 toxin [31]. Hence, the current study aims to evaluate the efficacy of the MR agent to reduce the adverse effects of moderate concentrations of AFB1 and T-2 toxin, individually or combined, and to test its efficacy on the performance and health of broiler chickens fed an artificially contaminated diet. Residues of mycotoxins and their metabolites might be present in edible tissues of animals receiving mycotoxin-contaminated feeds, which could subsequently create public health problems. Thus, we tested the efficacy of MR to reduce residual concentrations of AFB1, T-2 toxin and their metabolites in the liver and muscles of broilers fed AFB1 and T-2 toxin.

## 2. Materials and Methods

### 2.1. Birds and Diets

The experiment was performed on a total of 96 one-day-old broilers of Cobb 500 provenance. The experiment was organized according to a group control system. Broilers were fed according to the Cobb 500 manual, using 3 feeds—starter, grower and finisher feeds—and all feeds met or exceeded the nutrient recommendations set by Cobb-Vantress [32]. For the purpose of this study, broilers were divided into 8 equal groups (*n* = 12):

E-I group was fed a regular broiler diet with the addition of AFB1 at a concentration of 0.1 mg/kg of feed.

E-II group was fed a regular broiler diet with the addition of AFB1 at a concentration of 0.1 mg/kg of feed with 0.2% of the examined novel detoxification agent MR.

E-III group was fed a regular broiler diet with T-2 toxin at a concentration of 0.5 mg/kg of feed.

E-IV group was fed a regular broiler diet with T-2 toxin at a concentration of 0.5 mg/kg of feed with 0.2% of the examined novel detoxification agent MR.

E-V group was fed a regular diet with AFB1 at a concentration of 0.1 mg/kg of feed and T-2 toxin at a concentration 0.5 mg/kg of feed.

E-VI group was fed a regular diet with AFB1 at a concentration of 0.1 mg/kg of feed, T-2 toxin at a concentration of 0.5 mg/kg of feed and 0.2% of the examined novel detoxification agent MR.

C group was fed a regular broiler diet without contamination and without toxins and 0.2% of MR.

MR group was fed a regular broiler diet with 0.2% of the novel detoxification agent MR.

#### Feed Preparation

For the preparation of contaminated feed, AFB1 was obtained from *Aspergillus parasiticus* culture CBS 123.62. One kg of corn was roughly ground in a Waring laboratory mill, weighed into a 2 L laboratory beaker, covered with aluminum foil and autoclaved at 121 °C for 30 min. After the substrate had cooled, 150 mL of sterile distilled water was added, and the substrate was stirred and allowed to stand for half an hour. Two Petri plates of *A. parasiticus* were added (cultured on potato dextrose agar for 15 days). The substrate was stirred, and the aluminum foil was perforated for aeration. The contaminated substrate was incubated in a thermostat for 15 days at 25 °C and stirred daily. After the incubation was complete, the contaminated substrate was autoclaved again at 121 °C for 30 min and then placed in an oven at 105 °C for 48 h. The substrate was then ground and analyzed for aflatoxin content by LC-MS/MS.

T-2 toxin originating from *Fusarium langsethiae* culture (Fe2391) was used to prepare contaminated feed. One kg of corn was roughly ground in a Waring laboratory mill, weighed into a 2 L laboratory beaker, covered with aluminum foil and autoclaved at 121 °C for 30 min. After the substrate had cooled, 150 mL of sterile distilled water was added, and the substrate was stirred and allowed to stand for half an hour. Four Petri plates of *F. langsethiae* (cultured on potato dextrose agar for 15 days) were added. The substrate was stirred, and the aluminum foil was perforated for aeration. The substrate was incubated for 10 days at 10 °C and stirred daily. After the incubation was complete, the contaminated substrate was autoclaved again at 121 °C for 30 min and then placed in an oven at 105 °C for 48 h. The substrate was then ground and analyzed for T-2 content by LC-MS/MS.

The novel multicomponent mycotoxin detoxifying agent (MR) contains physical adsorption components, comprising activated zeolite (modified zeolite-clinoptilolite) and Saccharomyces cerevisiae cell wall, and detoxifying agents, specifically the spores of *Bacillus subtilis* and *Bacillus licheniformis*, *Saccharomyces cerevisiae* cell wall and hepatoprotective agent silymarin. These ingredients have been optimized (tested in vitro) to have an optimal effect on animals.

### 2.2. Data Collection and Recording

During the six weeks of trial, the following parameters were monitored:

Animal health; body weight (BW), measured at the beginning of each trial week (1–6 weeks); feed consumption (daily/group); body weight gain (BWG); and feed conversion ratio (FCR).

After six weeks of testing, all animals were euthanized by means of the cervical dislocation method (permitted for poultry). Thereafter, a pathomorphological examination was performed, and tissue samples were collected for pathohistological analysis (duodenum, liver, heart and Bursa Fabricii).

For determination of the presence and content of AFB1 and T-2 toxin and their metabolites in liver and pectoral musculature, samples were taken at the end of the experiment and frozen at −20 °C. Determination of AFB1 and T-2 toxin and their metabolite content in the liver and pectoral musculature was performed using the liquid chromatography (LC) technique with MS/MS mass detection.

#### 2.2.1. AFB1 and T-2 Toxin and Their Metabolites Analysis

The analysis of AFB1, B2, G1, G2, M1, T-2 toxins and their metabolites in the breast muscle and liver was performed using Agilent 6460 LC-MS/MS, and the implemented conditions are shown in Table 1. The method was developed and validated in house (in communication for publication). The LOQ (µg/kg) for AFB1, AFB2, AFG1, AFG2 was 0.1 μg/kg, 0.2 μg/kg for T-2 and 1 μg/kg for HT-2 toxin. The method was run using internal standards (13C24) AFB1 CRM Biopure^TM^—(0.5 µg/mL) for aflatoxins and (13C24) T-2 toxin; and CRM Biopure^TM^—(25 µg/mL) for T-2/HT-2 toxins. The recovery of more than 75% was recorded for all toxins. The method was linear from 0.1 to 1.2 μg/kg for aflatoxins, 0.2 to 4.0 μg/kg for T-2 and 1 to 20 μg/kg for HT-2 toxin.

The tissue samples were finely ground and thoroughly mixed using a blender. A 2 g test portion was removed for analysis. The samples were then extracted using a 10 mL extraction mixture (80% acetonitrile: 15% water: 5% formic acid) and by shaking the mixture in an orbital shaker at 200 rpm for 1 h at room temperature. After extraction, this portion was centrifuged at 4200× *g* for 5 min, and 7 mL of supernatant was removed and placed in another conical tube. The samples were cleaned by adding 2.8 g of MgSO_4_ and 0.7 g of NaCl to the supernatant and then vortexed for 60 s. These tubes were centrifuged at 4200× *g* for 5 min. A 1 mL solution was removed from the supernatant and diluted with 250 µL of water. Further cleanup was performed on an EMR Captiva cartridge (no cartridge conditioning was required): 1.25 mL of supernatant was passed through the cartridge (by gravity) and collected into a 15 mL centrifuge tube. When all the extract had passed through the cartridge, 400 µL of the extraction solvent was added and collected in the same centrifuge tube. The extract in the evaporator was evaporated at 1500 rpm at 40 °C. Then, 500 µL of the evaporated sample was added to the solvent for reconstitution (50% acetonitrile: 50% water containing 0.1% formic acid) and vortexed well. The prepared samples were filtered across a nylon membrane syringe (pore size 0.22 µm), filtered into a glass vial and vortexed. The samples were analyzed by LC-MS/MS using analytical column Agilent ZORBAX Rapid Resolution (Agilent, Palo Alto, CA, USA) HD 2.1 × 50 mm 1.8 µm and ZORBAX Eclipse Plus C18, 2.1 mm, 1.8 µm, UHPLC guard column under the following conditions.

Determination of mycotoxin mass fraction:

C_Mycotoxin_ (µg/kg) = (C × Vr × V)/m

where

C—determined concentration of mycotoxin (ng/mL);
Vr—reconstitution volume (0.5) (mL);
V—acetonitrile volume (8 mL) in the extraction portion;
M—amount of sample (g).

#### 2.2.2. Histopathological Examinations in Tissues of Broiler Chicks

The tissue samples for histopathological examination taken from the intestine, liver, heart and *Bursa Fabricii* were fixed in 10% buffered formalin. After standard processing in an automated tissue processor, the tissue samples were embedded in paraffin blocks, and 5 µm thick sections were stained with hematoxylin and eosin (HE) [33]. The results of histochemical staining were analyzed by light microscope (BX51, Olympus Optical, Tokyo, Japan). Photographs were taken with an Olympus Color View III^®^ digital camera. The number of the same histopathological changes per group per organ was noted.

#### 2.2.3. Statistical Analysis

All results were statistically analyzed for differences between groups by an analysis of variance (ANOVA). The results were processed using Graph Pad Prism^®^ 5.0 software (Graph Pad Software Inc., San Diego, CA, USA). All values are expressed as the mean ± SE.

## 3. Results

### 3.1. The Effect of Novel Detoxification Agent MR on Growth Parameters of Broiler Chicken

The average weekly body weight (BW), average weight gain (BWG), and average feed intake (FI) and feed conversion ratio (FCR) of the eight experimental treatments recorded for birds across performance trials are shown in Table 2, Table 3, Table 4, Table 5 and Table 6, respectively. Overall, the average weekly BW, BWG, FI and FCR in each group during the experimental period showed a difference, being lowest at the beginning and highest in the 6th week of the experiment. There were no statistically significant differences between the experimental treatments regarding the starting (1 d) broiler BW (not shown) and the BW, BWG, FI and FCR ratio during the starter (1–21 d) and growing (21–35 d) period. In the finisher period (35–42 d), broiler growth performance increased significantly (*p* < 0.05) in the group of broilers fed feed supplemented with MR when compared with the groups consuming the diets containing AFB1 alone or in combination with T-2 toxin (E-I, E-III and E-V).

At the 42nd day of the trail, the experimental groups receiving AFB1 and/or T-2 toxin either singly (E-I, E-III) or in combination (E-V) had significantly (*p* < 0.05) lower BW and weight gain than the groups receiving the toxins combined with 0.2% of MR (E-II, E-IV and E-VI) or the C group. BW and weight gain were not significantly different between experimental groups fed AFB1 (E-I) and T-2 toxin (E-III) singly, except for the group receiving AFB1 and T-2 toxin combined (E-V). Nevertheless, BW and weight gain were lowest in the group receiving AFB_1_ and T-2 toxin combined (E-V). The average BW and weight gain of the chickens consuming the novel detoxification agent MR was not significantly different (*p* > 0.05) from the control (C). Supplementation of poultry feeds with 0.2% of MR resulted in 12.46%, 12.13% and 2.91% increases of BW compared with the groups fed AFB1 and T-2 toxin either singly or in combination, respectively. When compared with group C, the BW in the whole experimental period was reduced by 25.61% with AFB1 alone, by 25.82% with T-2 toxin alone and by 35.37% with AFB1 and T-2 toxin in combination. Feeding AFB1 alone at a concentration of 0.1 mg/kg of diet showed a 33.90% overall reduction in BW gain when compared to the control group. Diets containing T-2 toxin (0.5 mg/kg) caused a reduction of 33.26% in BW gain over the 42 days when compared to the control group, while AFB1 and T-2 toxin in combination caused a reduction of 49.17% in BW gain when compared to the control group. The addition of MR to the AFB1- or/and T-2 toxin-contaminated diet reduced the adverse effects by 4.28%, 12.72% and 11.64% for AFB1 alone, T-2 toxin alone and AFB1 and T-2 toxin in combination, respectively.

A similar pattern was found in terms of feed intake. Feed intake was significantly (*p* < 0.05) reduced in groups fed AFB1- and/or T-2-contaminated diets compared with the control group and the groups where the addition of 0.2% of MR was applied (Table 3). For broiler chickens fed control feed, feed intake was increased with the addition of 0.2% of MR. When compared with controls, feed intake from the 1st to the 42nd day was reduced by 5.62% with AFB1 alone, by 6.36% with T-2 toxin alone and by 20.25% with AFB1 and T-2 toxin in combination, indicating a significant synergistic interaction. The addition of 0.2% of MR diminished the adverse effects by 6.80%, 7.33% and 3.49% for AFB1 alone, T-2 toxin alone and AFB1 and T-2 toxin in combination, respectively. The FCR was significantly higher in groups fed AFB1 and T-2 alone (2.2) compared with the C group and the groups which received 0.2% of MR (1.8 and 2.1, respectively). This suggests that there were impacts of feeding AFB_1_ and/or T-2 toxin on these parameters. Moreover, in this study, a significant synergistic interaction between AFB1 (0.10 mg/kg) and T-2 toxin (0.5 mg/kg) was observed.

### 3.2. The Effect of MR on Pathomorphological Alterations in Target Organs

Histological changes were observed in all tissues of the treated groups fed mycotoxin-contaminated diets with or without the MR detoxification agent. The lesions observed in the tissue samples (intestine, liver, heart and Bursa Fabricii) from the broiler chicks fed the mycotoxin-contaminated diets singly or in combination had significantly higher scores than those samples from birds fed the control feed (Table 7, Table 8 and Table 9). The lesions observed in the examined tissue samples were mild to moderate and less frequent in broilers fed mycotoxin-contaminated diets supplemented with the detoxification agent MR. These findings clearly demonstrate that, in broiler chicks, MR significantly diminishes the toxic effects of AFB1 when present singly or in combination with T-2 toxin.

#### 3.2.1. Intestine/Duodenum

**Figure 1:**
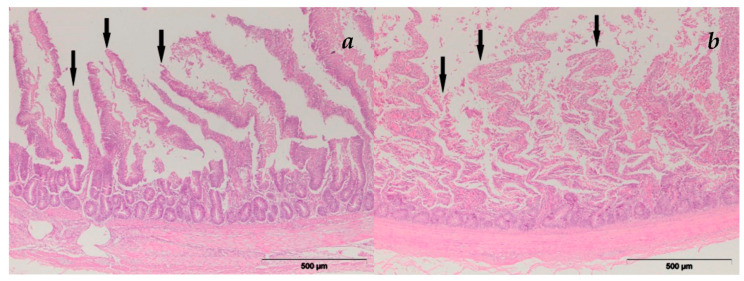
Intestinal villi atrophy by AFB1 and T-2 toxin combination, HE staining: (**a**) group E-V; (**b**) group E-VI (with MR).

The small intestinal histopathology results are presented in Figure 1. In the duodenum, changes were in the form of mucosal surface destruction and necrobiotic changes in the nuclei of the Lieberkühn crypt cells. Vascular changes were in the form of the overfilling of blood vessels with blood/hyperemia and/or accumulation of extravasate in the intestinal wall—hemorrhage. Destruction of the duodenal mucosa was characterized by the necrosis of enterocytes. Necrotic enterocytes were scattered along the surface of the villi. The mucus-producing goblet cells in the villous epithelium were hyperplasic and prominent due to distention caused by the mucus. Lieberkühn crypt cells showed signs of necrobiotic nuclear change—karyopicnosis.

#### 3.2.2. Liver

**Figure 2:**
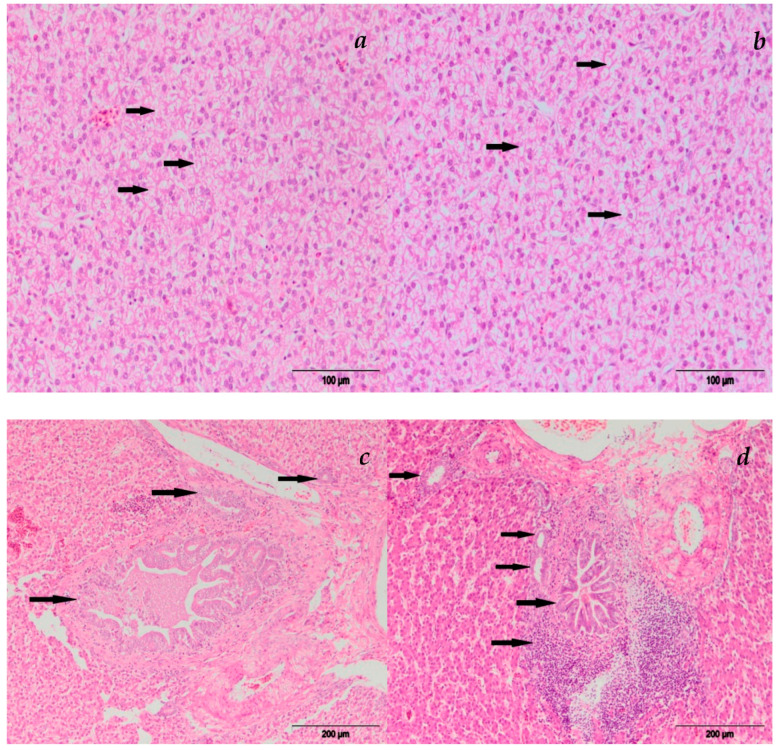
Liver HE staining of broiler chicks fed with diets containing AFB1 and T-2 toxin combination: (**a**) group E-III hydropic degeneration; (**b**) group E-IV hydropic degeneration; (**c**) group E-III cholangitis; and (**d**) group E-IV cholangitis and pericholangitis.

Histopathological examination revealed significant alterations in liver function in chickens fed the diet containing AFB1 alone or in combination with T-2 toxin (Figure 2). The main histological lesions observed in the liver were degenerative changes (cloudy swelling, vacuolar degeneration, hydropic change), focal necrosis of hepatocytes as well as vascular changes (hyperemia and hemorrhage) and infiltration of eosinophil granulocytes. The affected hepatocytes were swollen and pale, with cytoplasm which had a slightly granulated appearance and unclear nuclei. Hepatocytes with marked cloudy swelling compressed hepatic sinusoids. Vacuolar degeneration of hepatocytes was characterized by cellular swelling and a finely vacuolated appearance. An extreme variant of degenerative change of hepatocytes was in the form of hydropic change (ballooning degeneration), and it was characterized by the formation of large vacuoles and complete destruction of the cell. Necrotic hepatocytes were swollen with nuclear necrobiotic changes (karyopicnosis, karyorrhexis and karyolysis), followed by the rupture and fragmentation of cells. Hepatic hyperemia was characterized by the dilatation of hepatic sinusoids which were overfilled with blood. Hepatic hemorrhages were characterized by the accumulation of blood beneath the liver capsule and in the liver parenchyma. Focal accumulation of eosinophils was also noted in the liver parenchyma.

Hyperplasia of bile ductules in portal tracts consisted of proliferation of epithelial bile duct cells with the thickening of the ductal wall and narrowing of the ductal lumens. Inflammation of the biliary ducts—cholangitis—was in the form of destructive-desquamative cholangitis which was characterized by necrosis and desquamation of the bile duct epithelium. Pericholangitis—inflammation characterized by the infiltration of inflammatory cells—was often present around the areas of biliary destruction. Adding MR to the diet containing AFB1 alone or in combination with T-2 diminished the effects of the toxins on liver function.

#### 3.2.3. Heart

**Figure 3:**
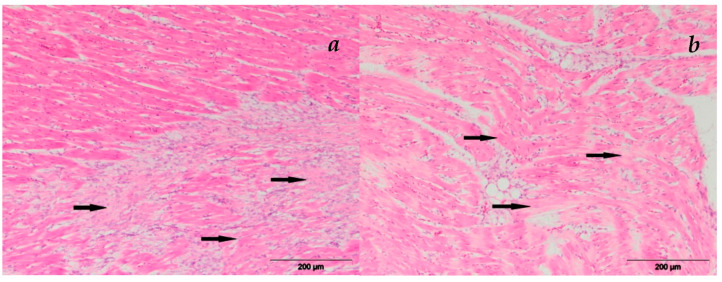
Heart myocardial degeneration, HE staining: (**a**) group E-V; (**b**) group E-VI.

Histopathological examination of the heart revealed focal infiltration of mononuclear cells and myocardial hemorrhages. Focal infiltration of mononuclear inflammatory cells located between the myocytes was noted. Myocardial hemorrhages were present, and they were characterized by the accumulation of erythrocytes between the myocytes. These histological changes were also observed in the groups experimentally treated with contaminated feed supplemented with MR, but these were mild and less frequent. In Figure 3, the degeneration of the myocard is shown.

#### 3.2.4. Bursa Fabricii

**Figure 4:**
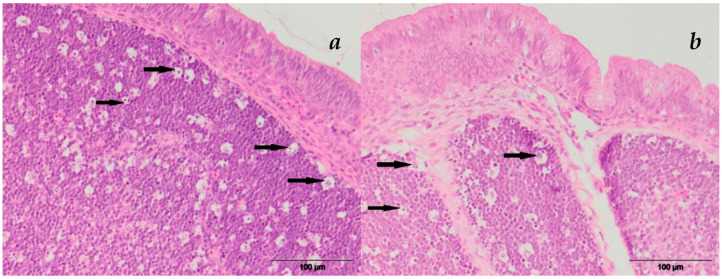
The apoptosis of *Bursa Fabricii*, HE staining: (**a**) group E-V; (**b**) group E-VI.

Bursal changes were in the form of apoptotic and necrotic changes of the lymphocytes within the follicles. Apoptotic changes were characterized by shrinking of the lymphocytes, which have a hypereosinophilic cytoplasm, are surrounded by a clear halo and often had numerous apoptotic bodies (Figure 4). Necrosis of the lymphocytes was characterized by cellular swelling with necrobiotic changes—karyopicnosis, karyorrhexis or karyolysis—and sometimes abundant eosinophilic cellular debris.

### 3.3. Residual Levels of AFB1/T-2 Toxin and Their Metabolites in Broilers’ Tissues

**Figure 5:**
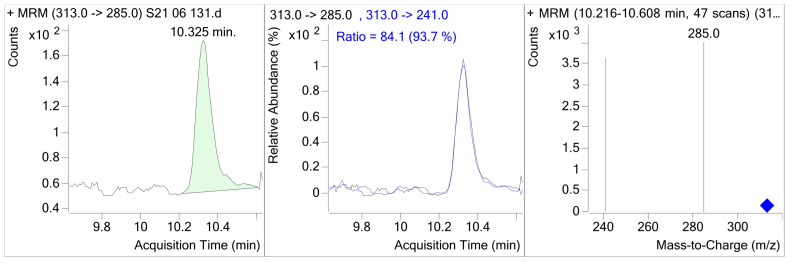
LC-MS/MS selected ion monitoring (SRM) chromatogram of the contaminated liver samples containing 0.12 μg/kg AFB1.

The data presented in Table 10 show that broilers fed 0.1 mg of AFB1/kg of feed (E-I) had average hepatic AFB1 concentrations of 0.235 μg/kg. Adding MR to the AFB1 diet (E-II) was efficacious in preventing the absorption of AFB1 in broilers. The dietary addition of 0.2% of MR (E-II) reduced the AFB1 concentration in liver samples by 51.06% (0.12 μg/kg). Therefore, AFB1 residual levels in broiler chicken livers fed diets with AFB1 plus the addition of MR (E-II) were significantly lower (*p* < 0.05) than of those that received AFB1 alone (E-I). Broilers from group E-V were fed AFB1 in combination with T-2 toxin, and group E-VI was fed the same combination with the addition of MR. Groups with and without MR (E-V and E-VI) did not show detectable residual levels of AFB1_,_ probably because they consumed significantly smaller amounts of feed. Regarding toxin concentrations in broiler muscles in 48 of the broilers fed AFB1_,_ either alone or in combination with T-2 and with and without MR, the concentration of AFB1 in muscles was below the detection limit. The results show that the feed–liver AFB1 transmission ratio was approximately 425:1 (0.235%). In Figure 5, the LC-MS/MS selected ion monitoring (SRM) chromatogram of the contaminated liver samples containing 0.120 μg/kg AFB1 is shown.

There were no detectable residues of T-2/HT-2 toxin or its metabolites, T-2 tetraol and T-2 triol, in the liver and muscle samples of broilers fed diets containing T-2 toxin. Information on carryover of contaminants from feed to animal food products is essential for appropriate human risk assessment of feed contaminants [34]. The present study highlights possible antagonistic or synergistic effects, which must be taken into account when conducting toxicological studies to evaluate the potential effect of different mixtures of toxins.

## 4. Discussion

### 4.1. The Effect of MR on Growth Parameters of Broiler Chicken

This study showed that in groups of broilers fed feed with AFB1 and T-2 toxin individually and in combination, there was a significant decrease in BW and BWG in proportion to the amount of toxin ingested. Group E-V, which received feed with AFB1, had the worst production results, which were 49.17% lower than the control group. The addition of 0.2% of MR to feed led to an improvement in production results (as described in the results), but by the end of the study, there was no complete restitution. In addition, AFB1 and T-2 toxin led to a significant reduction in feed intake (FI) as well as an increase in the feed conversion ratio (FCR). The conversion values were highest in the groups receiving AFB1 and T-2 toxin together with feed. The addition of 0.2% of MR led to a slight improvement. AFB1 molecules, as liposoluble compounds, easily pass through membranes, and upon entry into the cell, AFB1 and its metabolites can bind to a variety of molecules, the most important of which are DNA and RNA molecules, as well as proteins, and thus, their normal function is altered. The binding to DNA interrupts the transcription process, which leads to inhibition of the synthesis of normal proteins in the cell [35]. This is probably one of the mechanisms by which BW and BWG disorders occur. To our knowledge, authors who used similar amounts of toxins, but not the same detoxifiers (although they share some of the active components as the one we used), reported a decline in production results in treated animals, as well as a partial protective effect of mycotoxin detoxifiers [36,37].

### 4.2. The Effect of MR on Pathohistological (PH) Alterations in Target Organs

The results of our study related to PH changes in target organs are in agreement with authors who used similar doses of AFB1 and T-2 toxin in their experiments.

The addition of 0.2% of MR partially reduced the degree of PH changes in the target organs. The addition of 0.2% of MR could not completely neutralize the PH change. Our results are consistent with the reports of authors who used similar doses of mycotoxins and mycotoxin adsorbents in their experiments [38,39]. Since both AFB1 and T-2 toxin are primarily hepatotoxic, PH changes in the liver, but also in the heart and lymph tissues, are expected and are related to the metabolism and biotransformation of the toxin [40].

### 4.3. Residual Levels of AFB1 and T-2 Toxin and Their Metabolites in Broilers’ Tissues

Aflatoxin [35] and T-2 toxin [41] in particular are important for the poultry industry because of their toxicity, frequency of occurrence in feedstuffs and resultant economic losses. Besides the toxic effects, mycotoxin residues in poultry products may represent a threat to human health through the food chain [42]. Therefore, preventing mycotoxins from entering the food chain is imperative. In this study, the efficacy of the novel mycotoxin detoxification agent (MR) to detoxify poultry feeds contaminated with AFB1 and T-2 toxin, either singly or in combination, and consequently to reduce their distribution in the liver and muscles of the broiler chicks fed from the 1st to 42nd day has been evaluated. The addition of aflatoxin impairs all important performance parameters; this study confirmed the harmful effects of aflatoxin on the liver tissue of broilers, as well the fact that the liver is the principal site of accumulation of AFB1. Aflatoxins are considered extremely toxic due to their fast absorption in the gut and slow excretion [43]. Therefore, AFB1 residues were detected in various tissues, mainly in the liver, but also in the leg and breast muscles [44,45].

The current results indicate that the residue level of AFB1 was very low in the liver and that there were no residues in the muscles. This could be explained by the fact that in this study, low to moderate concentrations of AFB1 were trialed.

There were no detectable AFM1 residues in any of the tissues from the birds in the experimental groups. This is probably because of the higher polarity and increased water solubility of AFM1, which are more easily eliminated from tissues than the unmetabolized AFB1. Based on the low levels of AFB1 detected in the chicken tissues, no significant health risk was observed in the study. In terms of potential significance for human food, the results obtained from this study are consistent with those observed in the study by Hussain et al. [46], who reported that 28-day exposure of birds to contaminated diets at levels of 0.20 mg/kg did not pose a public health risk. However, dietary AFB1 concentrations above 0.40 mg/kg resulted in residual levels in the liver beyond the permissible limit for human consumption [47]. In that study, AFB1 muscle residues dropped below the permissible limit within 3 days of withdrawal of dietary AFB1. Similarly, Zaghini et al. [48] reported that the residue of AFB1 in livers and gizzards, as well as other edible tissues, occurred when diets were contaminated with AFB1 levels between 2.5 and 20 mg/kg, with increases in AFB1 in the diet resulting in higher residue levels in animal tissues [45]. In the study carried out by Hussain et al. [49], AFB1 residues in the liver were detected on the 3rd day of the trial in broilers that were fed 1600 ng/g of AFB1-contaminated feed. In the same study noted above, broiler chickens exposed to 6400 μg/kg of AFB1 had up to 6.97 and 3.27 ng/g of AFB1 residues in the liver and muscles, respectively. These residue levels were still detected for a longer duration in the younger birds, even after toxin withdrawal, which is an indication of slow elimination.

In this study, T-2 toxin or its metabolite residues were not observed in the liver and muscles of broilers. These results are consistent with the finding that T-2 toxin is rapidly metabolized and eliminated in different animal species, without accumulation in any tissue, and only traces of the toxins are found 24 h after oral or intravenous (iv) exposure to T-2 toxin [24]. Yang et al. [50] reported that T-2 toxin was rapidly metabolized into its derivatives, T-2 triol and HT-2 toxin, 6 h after the oral administration of 3 mg of T-2/kg BW, and only trace amounts of T-2 were detected, whereas large amounts of HT-2 were quantified in the muscles and liver. In contrast to the abovementioned studies or earlier studies, the reason for this discrepancy could be attributed to differences in the administered dosage, age of the animals and sensitivity of analytical methods used in this study.

Even though it does not bind to all the mycotoxins, this study confirmed that the addition of 0.2% of MR to the broiler feed contaminated with 0.1 mg/kg of AFB1 can effectively adsorb AFB1 and consequently reduce residual AFB1 and T-2/HT-2 toxins in edible poultry tissues. These results are consistent with the findings that the application of clinoptilolite at a rate of 0.2% in poultry feed under field conditions has achieved good results in the prevention of mycotoxins [51]. Moreover, these results suggest that in order to avoid the presence of residue in tissues or animal products, control strategies and continuous monitoring of mycotoxin in feed should be carried out.

## 5. Conclusions

This study included the examination of the negative effects of AFB1 and T-2 toxin, individually and in combination, on production results, pathohistological changes in target organs and the presence of residues of these mycotoxins and their metabolites in edible broiler tissues. At the same time, the possibility of the multicomponent detoxification preparation, added to broiler feed, exhibiting a protective effect was investigated. Observing the production results, we can conclude that aflatoxin and T-2 toxin individually had a detrimental effect on TM, growth, consumption and food conversion. Groups of broilers that received AFB1 and T-2 toxin together with feed achieved the worst production results. On the other hand, the broilers that received 0.2% of detoxification preparations with feed in addition to mycotoxins showed a partial improvement of production results, which can be attributed to the MR preparation for detoxification.

Observing the pathohistological changes in the target organs, we can conclude that the addition of 0.2% of MR to the diet reduced the number of PH changes in groups of broilers that received mycotoxins and a detoxification preparation.

A study of mycotoxin residues showed that they were detected in the liver of broilers that received 0.1 mg of AFB1 in their feed. Group E-II, which received 0.1 mg/kg of AFB1 and 0.2% of MR, showed 51.6% less AFB1 in the liver compared to group E-I. Broiler groups EV and E-VI, which received a combination of AFB1 and T-2 toxin, consumed significantly less feed compared to other experimental groups, which is why we believe that they ingested lower concentrations of AFB1 and that the residues in the liver were below the detection limit of the method. The fact that residues of T-2 toxin and its metabolites were not detected in edible broiler tissues is associated with the rapid biotransformation and elimination of T-2 toxin.

Finally, analyzing the effect of the detoxification preparation used in this study, we can conclude that it exhibits partially protective effects against the actions of AFB1 and T-2 toxin.

## Figures and Tables

**Table 1:** LC-MS/MS conditions.

Time [Min]	Mobile Phase A [%]	Mobile Phase B [%]	Flow [mL/Min]
0.00	88	12	0.2
5.00	88	12	0.2
5.01	50	50	0.2
16.00	0	100	0.2
17.00	0	100	0.2
17.01	88	12	0.2

**Table 2:** Average body weights (g) of broilers during the study ($\bar{x}$ ± Sx).

	Day 1	Day 7	Day 14	Day 21	Day 28	Day 35	Day 42
E-I	52.92 ± 0.73	130.8 ± 1.87 ^A^	339.5 ± 2.47 ^A^	627.8 ± 2.93 ^A^	935.8 ± 3.08 ^A^	1326.0 ± 7.52 ^A^	1826.0 ± 12.64 ^A^
E-II	51.58 ± 0.78	172.2 ± 2.7 ^B^	387.8 ± 2.21 ^A,B^	690.8 ± 2.61 ^A,B^	1032.0 ± 7.20 ^A,B^	1472.0 ± 2.81 ^A,B^	2055.0 ± 10.26 ^A,B^
E-III	51.83 ± 0.86	130.7 ± 1.75 ^A,C^	339.9 ± 2.69 ^A,C^	626.6 ± 3.77 ^A,C^	940.1 ± 3.51 ^A,C^	1326.0 ± 8.96 ^A,C^	1828.0 ± 13.31 ^A,C^
E-IV	51.58 ± 0.67	167.8 ± 2.15 ^B,D^	386.7 ± 2.23 ^A,B,D^	688.5 ± 2.71 ^A,B,D^	1034.0 ± 8.88 ^A,B,D^	1472.0 ± 3.57 ^A,B,D^	2054.0 ± 7.98 ^A,B,D^
E-V	51.58 ± 0.78	121.7 ± 2.0 ^A,C,E^	316.9 ± 1.61 ^A,B,C,D,E^	589.8 ± 4.35 ^A,B,C,D,E^	834.2 ± 3.63 ^A,B,C,D,E^	1215.0 ± 3.95 ^A,B,C,D,E^	1692.0 ± 1.91 ^A,B,C,D,E^
E-VI	51.50 ± 0.87	128.0 ± 1.85 ^A,C,E^	325.7 ± 3.68 ^A,b,C,d,E,F^	602.3 ± 2.86 ^A,B,C,D,E^	869.3 ± 3.34 ^A,B,C,D,E,F^	1255.0 ± 4.70 ^A,B,C,D,E,F^	1742.0 ± 4.91 ^A,B,C,D,E,f^
C	51.33 ± 0.89	173.9 ± 2.37	431.3 ± 2.46	791.2 ± 3.24	1235.0 ± 3.59	1808.0 ± 8.80	2336.0 ± 7.35
MR	51.33 ± 0.68	182.6 ± 2.58 ^B,C,D,E,F,G^	458.4 ± 2.72 ^A,B,C,D,E,F,G^	831.4 ± 2.50 ^A,B,C,D,E,F,G^	1304.0 ± 2.68 ^A,B,C,D,E,F,G^	1898.0 ± 3.03 ^A,B,C,D,E,F,G^	2542.0 ± 9.36 ^A,B,C,D,E,F,G^

The values with unlike superscripts differ at *p* < 0.01 (small letters) compared to group E-I—small b; E-III—small d; and E-V—small f. The values with unlike superscripts differ at *p* < 0.001 (capital letters) compared to group C—capital A; E-I—capital B; E-II—capital C; E-III—capital D; E-IV—capital E; E-V—capital F; and MR capital G.

**Table 3:** Average body weight gain (g) of broilers during the study ($\bar{x}$ ± Sx).

	Day 1–7	Day 7–14	Day 14–21	Day 21–28	Day 28–35	Day 35–42	Day 1–42
E-I	6.48 ± 0.17 ^A^	17.40 ± 0.11 ^A^	24.02 ± 0.24 ^A^	25.45 ± 0.33 ^A^	32.52 ± 0.61 ^A^	41.69 ± 1.26 ^A^	147.83 ± 1.06
E-II	10.05 ± 0.28 ^B^	17.96 ± 0.23 ^A^	25.25 ± 0.19 ^A^	28.42 ± 0.54 ^A,bb^	36.65 ± 0.61 ^A,B^	48.59 ± 0.91 ^aa,B^	154.15 ± 0.86
E-III	6.56 ± 0.15 ^A,C^	17.44 ± 0.25 ^A,d^	23.89 ± 0.40 ^A^	26.12 ± 0.52 ^A,c,^	32.15 ± 0.86 ^A,C^	41.8 ± 1.12 ^A,C^	148.0 ± 1.02
E-IV	9.68 ± 0.21 ^B,D^	18.24 ± 0.21 ^A^	25.15 ± 0.34 ^A^	28.75 ± 0.77 ^A,B,D^	36.53 ± 0.85 ^A,bb,D^	44.75 ± 0.77 ^aa,B,D^	166.83 ± 0.68
E-V	5.91 ± 0.25 ^A,C,E^	16.19 ± 0.25 ^A,C,E^	22.73 ± 0.39 ^A,C,E^	18.8 ± 0.46 ^A,B,C,D,E^	31.75 ± 0.46 ^A,C,E^	39.73 ± 0.28 ^A,C,E^	126.15 ± 0.14
E-VI	6.37 ± 0.13 ^A,C,E^	16.47 ± 0.36 ^A,cc,E^	23.05 ± 0.43 ^A,C,ee^	22.25 ± 0.34 ^A,B,C,D,E^	32.16 ± 0.39 ^A,C,E^	40.51 ± 0.70 ^A,C,E^	140.83 ± 0.39
C	10.21 ± 0.19	21.45 ± 0.33	29.98 ± 0.29	36.98 ± 0.39	47.73 ± 0.81	44.05 ± 1.03	189.08 ± 0.60
MR	10.94 ± 0.23 ^B,C, D,ee,F,G^	22.98 ± 0.35 ^B,C,D,E,F,G^	31.08 ± 0.32 ^B,C,D,E,F,G^	39.35 ± 0.31 ^a,B,C,D,E,F,G^	49.55 ± 0.39 ^B,C,D,E,F,G^	53.61 ± 0.70 ^A,B,D,F,G^	205.75 ± 0.80

The values with unlike superscripts differ at *p* < 0.05 (small letters) compared with group C—small a; E-II—small c; and E-III—small d. The values with unlike superscripts differ at *p* < 0.01 (double small letters) compared to group C—small aa; group E-I—small bb; E-II—small cc; and E-IV—small ee. The values with unlike superscripts differ at *p* < 0.001 (capital letters) compared to group C—capital A; E-I—capital B; E-II—capital C; E-III—capital D; E-IV—capital E; E-V—capital F; and MR capital G.

**Table 4:** The effects of 0.2% of MR on average daily feed intake (FI) of broiler chicks fed diets containing aflatoxin (0.1 mg/kg) or T-2 toxin (0.5 mg/kg), singly or in combination, during different periods of the study (g).

	Day 1–7	Day 7–14	Day 14–21	Day 21–28	Day 28–35	Day 35–42
E-I	45.23	69.59	81.88	103.88	133.72	122.27
E-II	47.58	70.07	82.28	117.76	151.58	131.71
E-III	47.64	63.53	76.48	101.85	139.83	128.76
E-IV	46.57	71.52	81.84	119.96	149.39	131.35
E-V	44.27	52.58	69.97	95.16	119.17	110.95
E-VI	45.42	57.79	71.61	95.33	120.94	115.88
C	47.82	73.67	86.32	110.92	141.83	126.97
MR	50.44	74.15	87.08	112.02	153.72	127.39

**Table 5:** The effects of 0.2% of MR on FCR of broiler chicks fed diets containing aflatoxin (0.1 mg/kg) or T-2 toxin (0.5 mg/kg), singly or in combination, during the study.

	FCR Day 1–21	FCR Day 21–42	FCR Day 1–42
E-I	2.4	2.1	2.2
E-II	2.2	2.0	2.1
E-III	2.3	2.1	2.2
E-IV	2.2	2.0	2.1
E-V	2.2	2.0	2.1
E-VI	2.2	2.0	2.1
C	1.9	1.7	1.8
MR	1.9	1.6	1.7

**Table 6:** Pearson’s correlation coefficient between average BW and average BWG within experimental group.

Group	BW	BWG	Correlation (r)
E-I	1826.0	147.83	0.857 *
E-II	2055	154.15	0.879 *
E-III	1828	148	0.857 *
E-IV	2054	166.83	0.858 *
E-V	1692	126.15	0.876 *
E-VI	1742	140.83	0.865 *
C	2336	189.08	0.817 *
MR	2542	205.75	0.845 *

Average BW (g), average BWG (g), * statistically significant (*p* < 0.05).

**Table 7:** The score of the PH changes of the duodenum of broilers submitted to AFB1 and T-2 toxin combination in relation to the numbers of animals examined.

PH Change	E-I	E-II	E-III	E-IV	E-V	E-VI
Hyperemia	6/12	2/12	11/12	5/12	6/11	2/12
Hemorrhage	7/12	2/12	3/12	2/12	5/11	1/12
Mucosal destruction and intestinal villi atrophy	12/12	9/12	8/12	4/12	9/11	3/12
Proliferation of goblet cells	10/12	4/12	3/12	0/12	6/11	4/12
Karyopicnosis of Lieberkühn crypts	8/12	0/12	8/12	2/12	8/11	1/12

**Table 8:** The score of the PH changes in liver of broilers chicks fed with diets containing AFB1 and T-2 toxin combination in relation to the numbers of animals examined.

Organ		PH Change	E-I	E-II	E-III	E-IV	E-V	E-VI
Liver	Hepatocytes	Cloudy swelling	5/12	2/12	10/12	6/12	7/11	5/12
		Vacuolar degeneration	9/12	6/12	5/12	4/12	7/11	1/12
		Hydrops degeneration	8/12	1/12	6/12	1/12	0/12	0/12
		Necrosis	3/12	2/12	7/12	4/12	2/11	2/12
	Bile ducts	Hyperplasia of bile ductules	6/12	4/12	6/12	2/12	7/11	4/12
		Desquamation of bile duct epithelium	8/12	7/12	7/12	4/12	9/11	5/12
		Pericholangitis	3/12	3/12	2/12	6/12	6/11	4/12
	**Interstitium**	Periportal fibrosis	3/12	1/12	1/12	0/12	2/12	0/12

**Table 9:** The score of the PH changes in the heart and *Bursa Fabricii* of broiler chicks fed with diets containing the AFB1 and T-2 toxin combination in relation to the numbers of animals examined.

Organ		PH Change	E-I	E-II	E-III	E-IV	E-V	E-VI
Heart	Myocardial cells	Degeneration	11/12	6/12	9/12	8/12	10/11	9/12
	Interstitium	Mononuclear cell infiltration	4/12	3/12	9/12	3/12	5/11	2/12
		Hemorrhage	6/12	6/12	8/12	6/12	4/11	3/12
*Bursa Fabricii*	Lymphoid follicles	Necrosis	7/12	1/12	12/12	7/12	2/11	2/12
		Apoptosis	9/12	5/12	6/12	6/12	11/11	5/12

**Table 10:** Concentrations of AFB1 toxin and their metabolites in broiler tissues in groups fed diets with the addition of AFB1 alone or simultaneously with T-2 toxin.

Tissue	E-I Group	E-II Group	E-V Group	E-VI Group
Liver	0.235 ± 0.07 ^aa^	0.12 ± 0.02 ^bb^	<LOD	<LOD
Muscle	<LOD	<LOD	<LOD	<LOD

LOD—limit of detection. The values with unlike superscripts differ at *p* < 0.01 (double small letters).

## Data Availability

Not applicable.
